# Searching for people with psychosis in the global south: mapping and establishing a case surveillance system in South Africa (PSYMAP-ZN study)

**DOI:** 10.1007/s00127-025-03011-1

**Published:** 2025-10-31

**Authors:** Jonathan K. Burns, Vuyokazi Ntlantsana, Tsatsawani Mkhombo, Saeeda Paruk, Lindokuhle Thela, Vidette Juby, Busisiwe Duba, Musa Sbiya, G. Nduku Wambua, Thirusha Naidu, Alex Cohen, Stefan du Plessis, Hans W. Hoek, James B. Kirkbride, Craig Morgan, Tessa Roberts, Ezra Susser, Leslie Swartz, Frank Tanser, Andrew Tomita, Wim Veling, Bonginkosi Chiliza

**Affiliations:** 1 University of KwaZulu-Natal, Durban, South Africa; 2 University of Exeter, Exeter, UK; 3 Africa Health Research Institute, Durban, South Africa; 4 University of Ottawa, Ottawa, Canada; 5 Cambridge University, Cambridge, UK; 6 London School of Hygiene & Tropical Medicine, London, UK; 7 Stellenbosch University, Stellenbosch, South Africa; 8 Parnassia Psychiatric Institute, The Hague, Netherlands; 9 University Medical Centre Groningen, University of Groningen, Groningen, Netherlands; 10 Columbia University, New York City, USA; 11 Division of Psychiatry, UCL, London, UK; 12 Health Service and Population Research Department, Institute of Psychiatry, Psychology & Neuroscience, King’s College London, London, UK; 13 ESRC Centre for Society and Mental Health, Institute of Psychiatry, Psychology and Neuroscience, King’s College London, London, UK; 14 Wolfson Institute of Population Health, Faculty of Medicine and Dentistry, Queen Mary University of London, London, UK

**Keywords:** Case finding, Psychosis, Mapping, Incidence, South Africa

## Abstract

**Purpose:**

Relatively little epidemiological evidence on psychosis from diverse settings in the Global South exists, where many people with untreated psychosis seek help outside of formal health service settings. Here, we report a preliminary mapping study of formal and informal community resources within a catchment area in South Africa that established an infrastructure that could be used to detect a representative sample of individuals with untreated psychosis.

**Methods:**

PSYMAP-ZN is a 3-year study of incidence, clinical presentation and associated risk factors for untreated psychosis in Msunduzi Municipality in South Africa. We conducted a preliminary mapping study of the region in which we aimed to document all potential providers of care (gatekeepers) in both formal (health services) and informal (folk) sectors, with the purpose of enrolling them in a collaborative case surveillance system. We drew on official sources, local knowledge from key stakeholders and utilised snowballing techniques.

**Results:**

We established a surveillance system which included (a) all secondary mental health and primary care services (b) the majority of informal providers (including traditional health practitioners, religious institutions) and (c) a wide range of key informants.

**Conclusion:**

Expanding the global knowledge base on psychosis to diverse settings in the Global South requires a surveillance and case-detection method that includes (in addition to formal health settings) informal settings and local key informant knowledge in the community. This preliminary ‘mapping’ process established a platform for the ongoing PSYMAP study of untreated psychosis in South Africa.

## Introduction

The epidemiology of psychosis is dominated by evidence from the Global North, where most knowledge production on incidence, risk factors, presentation, and course and outcome of psychosis originates [1]. This is problematic as the lack of evidence from the Global South makes for an incomplete understanding of psychotic disorders, and hampers efforts to develop appropriate and effective services for those in need [2].

It is now well established that there is replicable and predictable heterogeneity in the incidence of psychosis, including by age, sex and ethnicity but notably, almost all this evidence comes from the high-income Global North [1, 3, 4]. In addition, evidence from the Global North suggests that several environmental risk factors are associated with psychosis risk, such as early childhood experiences of trauma, migration, urban upbringing and the impact of the structural, social and economic environment [5, 6]. This has received insufficient attention in diverse low- and middle-income country (LMIC) settings in the Global South, where many regions are characterised by high levels of poverty, inequality, interpersonal violence, food insecurity, infectious diseases such as HIV, as well as limited availability and access to mental health services [7, 8]. An updated review highlighted “the dearth of robust evidence on the incidence of psychotic disorders in LMICs” [3]; but notably the INTREPID study has recently published rates of untreated psychosis for 3 sites in India, Nigeria and Trinidad [9].

Such research in LMIC settings is challenging because most epidemiological studies in the Global North rely on case ascertainment via formal healthcare settings based on the assumption that the vast majority of individuals becoming ill with psychosis for the first time will present to or come to the notice of mental health services [1, 10]. This assumption is likely to be more plausible in the Global North – and particularly in settings with universal health care coverage – where, generally speaking, the availability of and access to mental health services is relatively good.

The situation, however, in most regions of the Global South is different and such assumptions will rarely hold. Both research and public health efforts require a different form of case surveillance system that is underpinned by the key principle of intersectoral collaboration. The ‘treatment gap’ (i.e. the gap between need and availability and access to specialist services) is a notable challenge in many parts of Africa, Asia and Latin America and the Caribbean [11, 12]. An exception is Chile which compares favourably with European countries in first episode psychosis (FEP) ascertainment. In South Africa, the mental health gap has been estimated to be around 80%, while the World Health Organisation have reported that two-thirds of patients with schizophrenia in 50 LMICs did not have access to specialised mental health care [12, 13]. Furthermore, the median duration of untreated psychosis has been estimated to be longer in Africa (23 weeks) and Asia (17 weeks) than in Europe (12 weeks) and Australasia (8 weeks) [14].

Morgan and colleagues [10] cite Kleinman’s model of health care services as “a useful framework for formalising approaches to identifying cases of psychosis across diverse settings.” Kleinman outlines three sectors in which illness is managed: the professional (medical) sector; the folk (informal, spiritual, traditional) sector; and the popular (self-care, family support, etc.) sector [15]. Thus, particularly where formal, professional services are unavailable or inaccessible, one needs to look within the informal folk and popular sectors for individuals with psychosis. This is borne out within the African context where there is good evidence showing that a large proportion of people with early psychosis first consult traditional or spiritual healers before making contact with formal health services [16] and often continue to consult healers during and after this contact [17]. Nonetheless, almost all incidence studies of psychosis conducted in the Global South to date have based their case-finding on presentation of individuals at formal health facilities – hospitals and clinics (Table 1; [9, 18–37]), with the exception of the recent INTREPID study.

**Table 1:** Case-finding methods in previous incident studies in the global South

Author	Country	Diagnosis	Number	Case-finding methods	Incidence*
Bhugra et al., 1996	Trinidad	any psychosis	56	hospital in- and out- and CMHS	26
Binbay et al., 2024	Turkey	any psychosis	115	Hospital, family medicine and health directorate registries	38.5
		NAP	62	Hospital, family medicine and health directorate registries	20.7
Burns & Esterhuizen, 2008	South Africa	any psychosis	160	hospital admissions from records	31.5
Caetano, 1981	Brazil	schizophrenia	178,173	hospital inpatient records	26
Chen, 1984	China	schizophrenia	34	household survey	11.5
da Rocha, 2021	Brazil	NAP	1,549,298	first admission hospital records	82.9
Del Ben, 2019	Brazil	any psychosis	588	first contact with hospital and community MH services plus leakage	19.1
Gonzalez-Valderrama, 2022	Chile	NAP	32,358	national register data	18.9
Handal, 1997	Costa Rica	schizophrenia	2934	first admission, hospital records	48.2
Hanoeman, 2002	Suriname	NAP	73	first admission	16
Hickling & Rogers-Johnson, 1995	Jamaica	NAP	320	out-patient clinics and community MH services	23.6
		schizophrenia	285	out-patient clinics and community MH services	20.9
Huang, 1990	China	schizophrenia	1359	first admission	24.9
Ihezue, 1982	Nigeria	schizophrenia	67	first admission	14
Jablensky et al., 1992	India	NAP	406	formal MH and informal services (healers, informants)	31.8
	USSR	schizophrenia	136	formal MH and informal services (healers, informants)	10.7
Jongsma, 2018	Brazil	any psychosis	565	case-finding from MH services	21.5
		NAP	389	case-finding from MH services	14.8
Menezes, 2007	Brazil	any psychosis	367	case finding with leakage from MH services	15.9
		NAP	231	case finding with leakage from MH services	10
Morgan, 2023	India	any psychosis	268	formal and informal case-finding with leakage	20.7
	Nigeria	any psychosis	196	formal and informal case-finding with leakage	14.4
	Trinidad	any psychosis	574	formal and informal case-finding with leakage	59.1
Rajkiumar, 1993	India	schizophrenia	15	case-finding in the community plus leakage	35
Selten, 2005	Suriname	NAP	64	hospital and primary care	16.8
Song, 2022	Colombia	schizophrenia	1053	hospital admissions from records	45
Wig, 1993	India	schizophrenia	209	first contact with health services and key informants (not THPs/faith)	39

*Incidence is per 100 000 population at risk

NAP = Non-affective psychosis

The INTREPID study set out to search for people with untreated psychosis, not just within mental health services, but also within the informal (folk) and popular sectors [2]. For these purposes, “untreated” was operationalised as “not continuously using antipsychotic medication for 30 days prior to the start of the study” and rates of untreated psychosis was used as a proxy for incidence. Case-finding methods were designed to detect representative samples (and as near to epidemiologically complete samples as possible in such settings) in catchment areas in India, Nigeria, and Trinidad, and were based on extensive preparatory work that sought to ‘map’ and engage many potential providers of care across all sectors, where individuals with untreated psychosis may present or be detected. Based on these mapping exercises, case-finding was conducted in both formal (health service) and informal (folk) settings, and with the additional use of key informants embedded within the communities [10]. INTREPID has recently published rates of untreated psychosis for all three sites [9], demonstrating that their “methodological template that can be adapted as a basis for the generation of representative samples of psychosis in other settings” [10] is indeed able to estimate incidence rates in such settings.

The aim of the present paper is to describe the methodology and findings of a mapping study that sought to adopt and adapt the methodological template of the INTREPID study in the context of a mixed rural, peri-urban and urban catchment area in part of KwaZulu-Natal Province in South Africa. The learning provided by this vital preparatory study allowed us to subsequently establish a three-year epidemiological study of untreated psychosis, known as the PSYMAP-ZN study, in a catchment area (Msunduzi Municipality) in one part of KwaZulu-Natal Province, South Africa. The main aims of the PSYMAP-ZN study were to determine the incidence and clinical presentation of, and associated risk factors for, untreated psychosis in this setting.

**Fig. 1:**
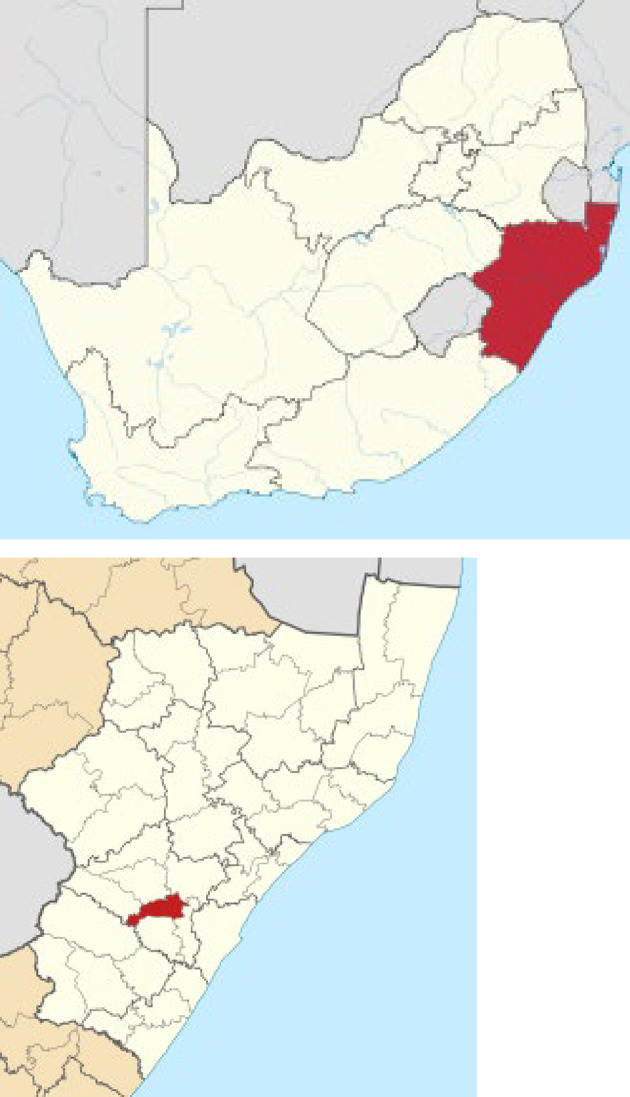
Maps 1–2: Location of Msunduzi Municipality within South Africa Map 1: Map of South Africa showing KwaZulu-Natal Province Map 2: Map of KwaZulu-Natal Province showing Msunduzi Municipality

In the current paper, we report on a preparatory mapping study, building on earlier pilot work in the Vulindlela subregion of Msunduzi from the INCET study [17, 38], which we expanded to the whole catchment area through the first 12 months of PSYMAP-ZN. We had successfully piloted a collaboration with traditional health practitioners (THPs) in Vulindlela during INCET. We had not, however, mapped other providers in Vulindlela, nor in the other parts of Msunduzi making this a key aim of the initial phase of PSYMAP-ZN. Specifically, the purpose of the mapping study was to identify, document and engage with potential providers of care throughout Msunduzi in both formal and informal sectors, as well as key informants within the community, to set up an infrastructure that could be used both to identify a representative sample of individuals with untreated psychosis and to provide a model for a public health approach to the surveillance of and collaborative care of people with psychosis in such settings (Fig. 1).

## Methods

In designing the PSYMAP-ZN study, we adapted the methodology of the INTREPID study [2, 10] to facilitate comparable, transparent and harmonized methods and results for studies of psychosis incidence in the Global South.

### Catchment area

**Fig. 2:**
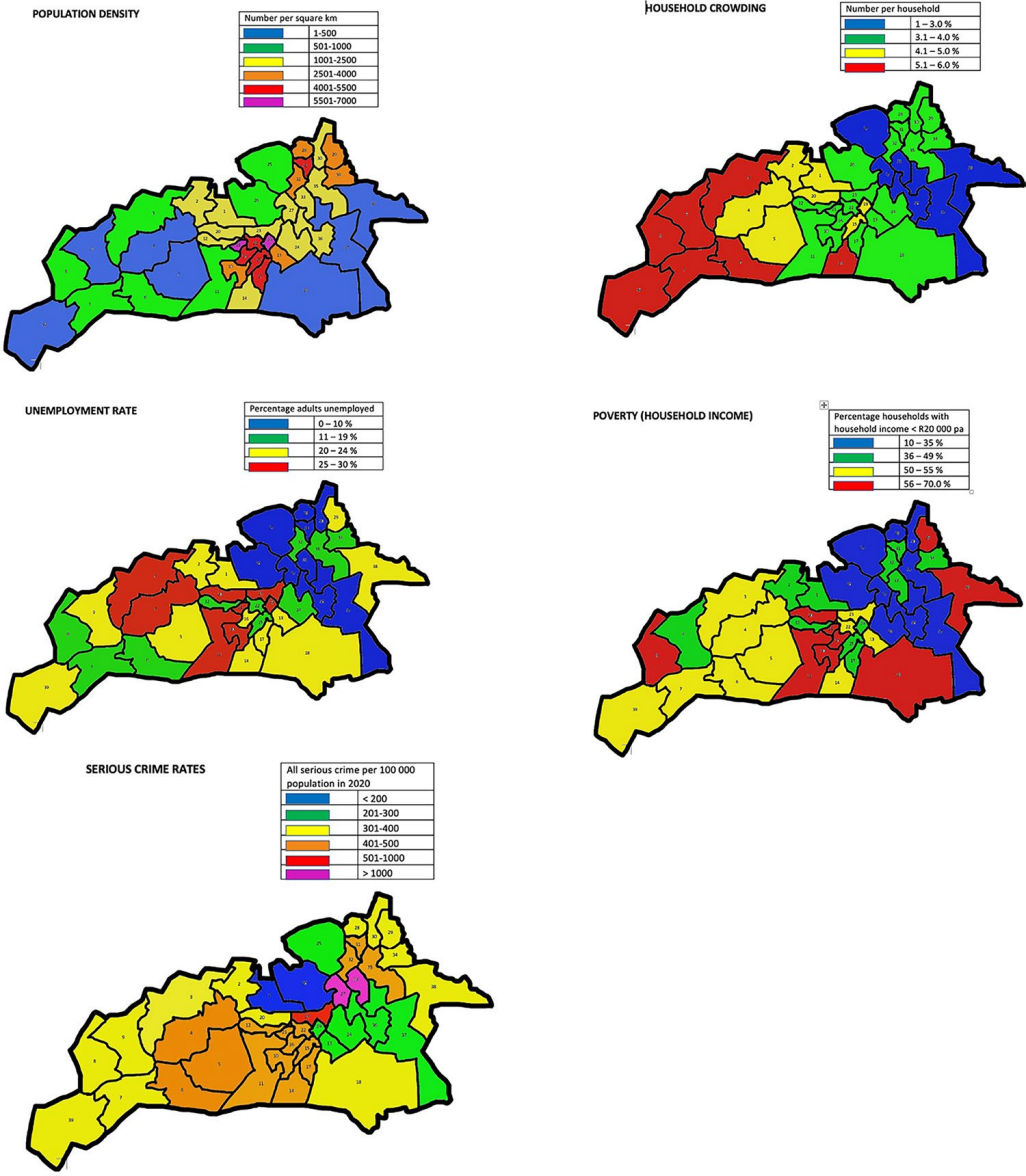
Maps 3–7: Household crowding, unemployment, poverty, crime rates for Msunduzi

Our catchment area was Msunduzi Municipality, within the District of Umgungundlovu in KwaZulu-Natal Province, South Africa (**Maps 1–2**). It comprises 39 electoral wards. Msunduzi is a region 100 km inland of the major port city of Durban and comprises an area of 634 square kilometers. Msunduzi has urban, peri-urban and rural regions – the city of Pietermaritzburg, Edendale township and the rural Vulindlela area respectively. Vulindlela is under the local administration of traditional authorities and was the site of our pilot study, INCET [17, 38]. Across Msunduzi Municipality, one finds considerable environmental and socioeconomic differences. Some wealthy neighbourhoods have large expensive homes and gardens, low crime rates, excellent health statistics and a high quality of life. Other neighbourhoods comprise densely populated low-cost housing and shacks, with high household occupancy, low employment rates, poor life expectancy and some of the highest rates of crime in the country. Msunduzi also has amongst the highest prevalence of HIV in South Africa. The population of Msunduzi estimated in the 2022 Census was 817 725 [39]. For further demographic, socioeconomic, crime and health data on Msunduzi, see Fig. 2 (Maps 3–7) and Table 2.

**Table 2:** Demographics, socio-economic, crime and health data for Msunduzi

		Percentage	Number	
Demographics				
	Total population		817 725	
	Population density		1 089 per square km	
Age	Population aged 18–64 Median age	58	474 280	24 years
Gender	Male Female	47.7 52.3	390 308 427 417	
Ethnicity	Black Coloured Indian White Other	77.8 3.0 13.9 5.0 0.3	636 190 24 214 113 400 41 260 2 661	
Education	Tertiary Completed secondary Completed primary Less than primary	11 44 33 11		
Religion	Christian Traditional Hinduism Islam Non-religious	75 13.5 4.2 1.9 3.4		
**Socioeconomics**				
Number of households			180 469	
Persons per household			3.7	
Type of dwelling	Houses Traditional Shacks Flat in backyard Other	71.2 11.2 8.4 3.1 6.1	128 494 20 213 15 159 5 594 11 009	
Annual household income (Rands)	>600 000 300–600 000 150–300 000 75–150 000 40–75 000 20–40 000 10–20 000 5–10 000 < 5 000	3 6 9 10 12 16 16 7 21		
Mean annual household income (Rands)				29 400
Employment	Employed Unemployed Discouraged work seeker Other not economically active	36 18 6 40		
Poverty	Living below the poverty line	63		
Inequality	(Gini coefficient)			0.62
**Health**				
HIV	Population prevalence (15–49) Female Male	36.3 44.1 28		
Life expectancy (years)	Female Male			63.6 57.4
**Crime rates**				
			Number per annum	Rate per 100 000 pop.
Murder rate			511	71
Sexual crime rate			653	85
Assault GBH rates			1801	237

### Identifying and engaging with providers

Within Msunduzi, we sought to identify all potential providers within the formal health services (hospitals, clinics, etc.) and within the informal sector (i.e. traditional healers, spiritual and other community entities). Because of complexities that exist in the political administration of the region, we also needed to identify key stakeholders whose support was a prerequisite for undertaking the study in this region. Finally, we sought to identify any key informants within the community who might have local knowledge of or involvement with individuals with psychosis in their community and would likely have important knowledge and insights into the complex networks within those communities.

The identification and engagement with providers was, from the outset, a snowball process, whereby initial contact with individuals or organisations provided new information that led to the identification of potential new contacts. This process was determined to some degree by a requirement to first engage with (and obtain approval and support from) key leaders in the various sectors. Thus, for example, we had to obtain authorisation from regional formal health authorities, before engaging with primary health care and other health services. Having this authorisation also meant that health workers at these facilities would to some extent be ‘bound’ to cooperate with and support our study.

Similarly, an initial meeting with the local Inkosi (or regional traditional chief) and his Traditional Council was appropriate in terms of local practices before we could initiate contact with THPs in the region. This initial engagement served two important purposes, both of which were key to engagement with THPs working in the catchment area and had been made clear during our pilot study: (1) without formal permission from the Chief, THPs would not be permitted to engage and collaborate with us; (2) this cooperation with the Traditional Council meant that we were assisted by them in identifying *bona fide*, recognised THPs [40]. For context, it is important to explain some of the complexities related to the efforts of Traditional authorities in South Africa to recognise, register and accredit practising THPs as part of the health workforce in the country [41]. THPs play an important and respected role in many parts of South Africa, where they are reported to be consulted by approximately 70% of black South Africans [42]. But available research in KwaZulu-Natal has found that only a quarter of THPs in the province were registered with THP organisations [42]. There are many reasons why many THPs are not registered, so one cannot assume that all unregistered THPs are not offering good and ethical services. Based on estimates for the whole of KwaZulu-Natal Province [42], it is reasonable to estimate that there may be between 1500 and 2000 THPs working in Msunduzi. In PSYMAP-ZN we relied upon the local Traditional Council to place us in contact with registered THPs in the region (a subset of all THPs in the region). One final important point is that the harmful practices reportedly used by THPs in some regions of Africa are uncommon in this region [42].

Thus, our identification and engagement activities included the following individuals and structures:

1. The KwaZulu-Natal Department of Health, including the Mental Health, Primary Care and Hospital Programme Managers; programme leads at the District Health Office, including the District lead for Community Health Services; Hospital Managers and Clinical Heads; and Managers of Community Health Centres and Primary Health Care Clinics.
2. Psychiatrists, Psychologists, other mental health practitioners and General Practitioner groups in the private sector.
3. The KwaZulu-Natal Department of Social Development and Department of Cooperative Government and Traditional Affairs; Ward Committees and Ward Councillors; and Community Care Givers and their Supervisors.
4. The South African Police Services; local Christian, Muslim and Hindu religious leaders; the University of KwaZulu-Natal Student Affairs office; the Durban University of Technology and research organisations working within the catchment area.
5. The Inkosi (Traditional Chief) in the region and his Traditional Council of iziNdunas; regional THP organisational representatives; and THPs recognised by the THP organisations and operational in the catchment area.

In engaging with various providers and stakeholders, we prepared informational materials, based on extensive pilot work (see below), specific for different audiences (including translations in isiZulu), conducted meetings with key individuals and groups, and prepared referral forms, referral pathways and agreement on a system of referral and regular surveillance to identify individuals with possible psychosis presenting to (or known to) the providers. During our pilot study we had undertaken qualitative and ethnographic work with THPs in the Vulindlela sub-region, including sessions on both local understandings of mental health phenomena and expressions/idioms of distress, and medical concepts of psychopathology [17, 43]. We had co-developed with THPs a concept we termed ‘mentally disturbed’ as a description of the presentation we were interested in for our study [38]. We could thus utilise this approach that had proved successful in the pilot study [38], when engaging with THPs for the PSYMAP-ZN study.

We established a Community Advisory Group (CAG) with elected representatives from most of the above stakeholder groups. This CAG had a budget, a chairperson, developed standard operating procedures, were consulted on key issues and met quarterly for the duration of the study. As with the key informants, members of the CAG, by virtue of their being embedded within the community, were important contributors of knowledge and guidance in our ambition to understand the complex networks existing within these communities.

### Collation of data

For each provider and informant identified, we recorded the following information: name, contact details, location, services provided, staffing, numbers and types of patients seen (including for mental health/problems), public versus private, and costs to patients. We derived this information both from publicly available sources (e.g. websites) and from the providers and informants/stakeholders themselves.

## Findings

**Fig. 3:**
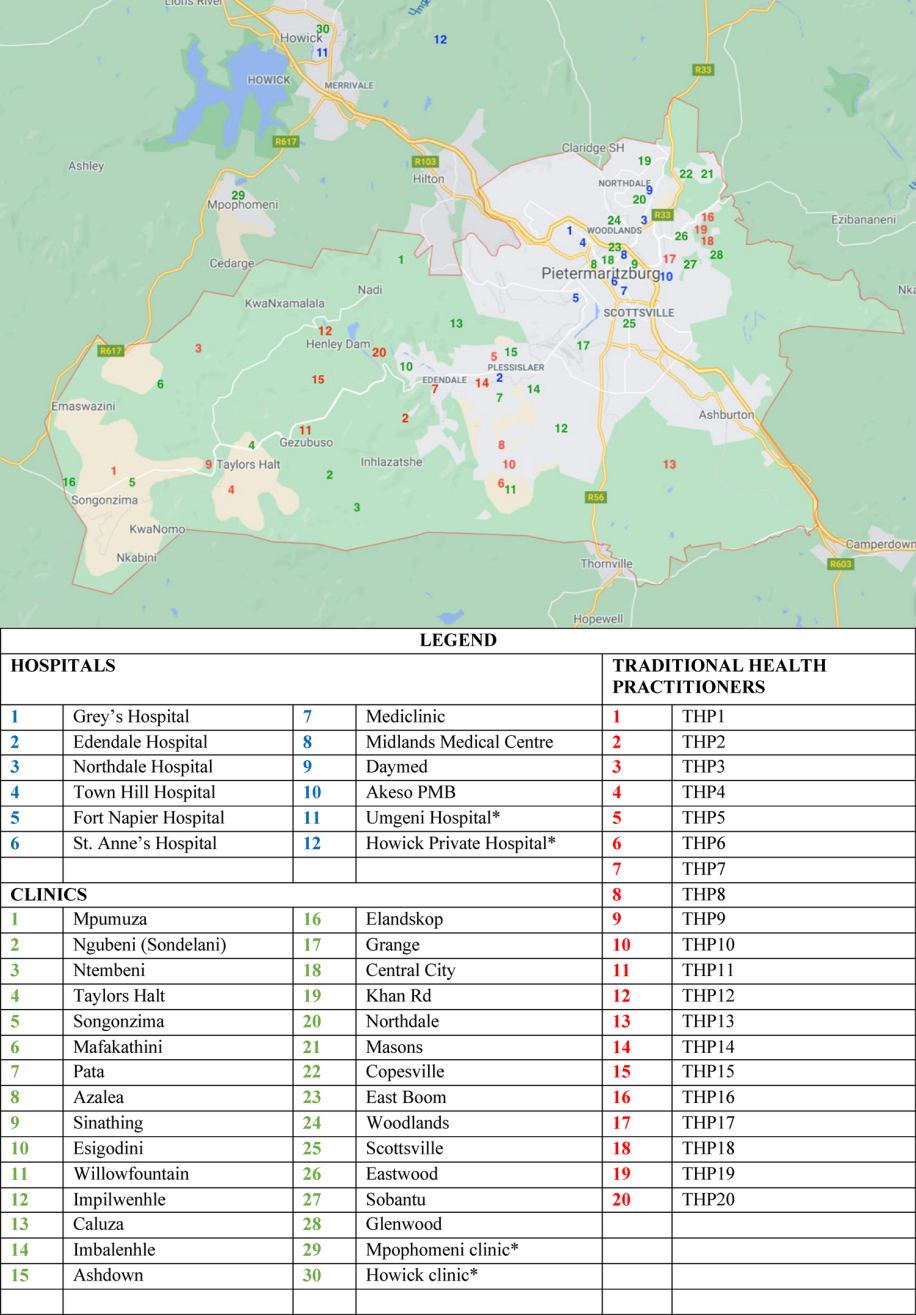
Map 8: Hospitals, clinics and key informant THPs in Msunduzi

During the mapping phase of PSYMAP-ZN, we identified a large number and variety of potential providers and informants from across the catchment area, from both formal and informal sectors (Fig. 3 (**Map 8**)). Using this information, we established a surveillance and detection system of services, providers and informants that we could use to monitor and screen for people with untreated or first-episode psychosis. Below we present a summary of these findings.

### Formal sector

The formal health sector in Msunduzi Municipality comprises public and private services. Public services consist of hospitals, with inpatient beds and outpatient clinics, and community health services which include community health centres (CHCs) and primary health care clinics (PHCs). All are publicly funded and are essentially free, although higher-income earners pay a means-tested rate for inpatient care.

Town Hill Hospital (THH), located in Msunduzi, is the main specialised public psychiatric hospital in the region, serving roughly one-third of KwaZulu-Natal Province (and an approximate population of 3 million). THH has 280 beds and is staffed by seven psychiatrists, 10 medical officers and six registrars, as well as psychologists, social workers and nurses. It provides some specialised services, such as child and adolescent services, old age services and psychotherapy services. Most patients admitted to THH are referred on discharge to community services local to their place of residence. There is also a specialised forensic psychiatry hospital within Msunduzi, Fort Napier Hospital, with 370 forensic beds. There are three general hospitals also within Msunduzi. Grey’s Hospital is the main regional tertiary referral hospital, with 500 beds, providing a wide range of specialised medical services including neuroimaging. It has no dedicated mental health beds, but psychiatrists from THH do provide some in-reach consultation-liaison services. Harry Gwala Regional Hospital (HGRH) is a large regional general hospital, located in the peri-urban section of Msunduzi. It is the fourth largest hospital in South Africa with 897 beds and has a dedicated psychiatric ward of 15 beds. HGRH currently has one psychiatrist who supervises care for approximately 95–105 in-patients at a time (with around 80–90 patients admitted in general medical wards) and runs outpatient clinics daily. Northdale Hospital is a district general hospital with 430 beds. There are no psychiatrists but have nine male psychiatric beds; patients with psychiatric disorders are also accommodated in general medical wards (approximately 50 at any time) and are managed by medical officers.

There are six private hospitals within Msunduzi, of which four provide outpatient psychiatric services, with one of these four, Akeso Hospital, providing 58 inpatient beds; these four hospitals are served by 12 psychiatrists. Private psychiatric services are only accessed by individuals who have private health insurance – in 2022 this was reported to be 15% of the South African population [44].

There are approximately 200 general practitioners (GPs) within the region – GPs receive basic training in mental health during their medical training and would refer the majority of patients with first-episode psychosis to a public health facility (PHC clinic or hospital) or to a private hospital in the area.

Community health services in Msunduzi comprise 30 public facilities (2 CHCs, 28 PHCs), located within 21 of the 39 electoral wards. All of these provide non-specialised general medical care, including mental health conditions, and are generally staffed by general nurses and health assistants, with visiting general practitioners. Nurses working in primary care receive training that includes material from WHO’s mhGAP guide [45], as well as the Standard Treatment Guidelines and Essential Medicines List published by the Department of Health [46].

Community health services are supported by a cadre of workers called Community Care Givers (CCGs), introduced in 2011 to support primary health care [47]. CCGs operate in small teams with a supervisor who is often a nurse. CCGs are allocated to electoral wards and are based at a clinic within the ward but spend much of their time conducting home visits where they provide basic maternal, child health and chronic care. Msunduzi has a total of 187 CCGs, allocated across 28 electoral wards. The CCGs were regarded as key informants for the purposes of the PSYMAP-ZN study since they are embedded in the community and often have long-standing knowledge and professional and social relationships with many of the households and their occupants.

### Informal sector

Msunduzi is a culturally diverse region with multiple traditional, spiritual and religious beliefs and practices adhered to by a substantial proportion of the population (see Table 2). As such, it was impossible to identify and engage with all practitioners across the region. We were able to identify approximately 50 Christian churches/groupings, 20 Muslim mosques and masjids and 10 Hindu temples/organisations, of which approximately 80% were engaged in the study for regular phone-calls from our team. About half of the Christian institutions are Catholic or Protestant denominational churches, while the other half are non-denominational churches, temples and independent (often Pentecostal) groupings. Many of the latter practice faith-based healing (including prayer, fasting and other rituals), and we believe we engaged most of these as key informants in our study. We identified only one institution (a Pentecostal church in Edendale) where people with mental (and physical) illnesses would actually reside for a period, receiving faith-based healing practices in preference to biomedical treatment. In this particular setting we were informed that restraints (including chaining) were used on some individuals, although we were not permitted access and our attempts at engagement were rejected. We notified local formal healthcare providers of this situation so that they could develop an appropriate strategy within the local sociocultural context in which this practice arose.

During our mapping phase of PSYMAP-ZN, we identified and met with many groups of THPs who had been invited to attend our meetings by local traditional leaders and counsellors. We believe THPs attending these meetings were selected based on several factors including: being recognised (registered) with THP organisations, their availability, and being known to treat people with mental health presentations. In terms of actual engagement in PSYMAP-ZN, we met with around 100 THPs who were informed about the study and were directed by local traditional authorities to contact us if they had clients who appeared “mentally disturbed”. Of these 100, we managed to engage 20 THPs from across the catchment area as key informants in our surveillance and monitoring system. Essentially, these 20 self-selected by volunteering to actively participate in the study, and thereafter they were contacted by our team every fortnight for the duration of our case ascertainment period. This group included both major categories of THP, namely izangoma (diviners who intercede with ancestral spirits) and izinyanga (herbalists who heal using plants, herbs and animal parts) [41]. Fifteen of these THP key informants provided both ‘inpatient’ and ‘outpatient’ care, while five only provided consultations only. Most THPs charged approximately R100 (£5) per consultation (see Table 3).

**Table 3:** Traditional health practitioners engaged in the study

	**Location - Ward**	**Services offered**	**No of beds (total)**	**No of users per month**	**No of MH users per month**	**Staff**	**Costs (Rands per consult)**	**Monitored**
THP 1	39	In-patients & Out-patients	5	10	1	3	100	YES
THP 2	5	Out-patients	0	30	1	1	100	YES
THP 3	9	Out-patients	0	5	2	1	100	YES
THP 4	6	In-patients & Out-patients	6	10	4	1	100	YES
THP 5	23	In-patients & Out-patients	3	12	3	3	100	YES
THP 6	14	Out-patients	0	10	1	1	50	YES
THP 7	21	Out-patients	0	20	5	2	250–340	YES
THP 8	10	In-patients & Out-patients	5	5	1	4	100	YES
THP 9	7	In-patients & Out-patients	5	10	2	3	100	YES
THP 10	14	Out-patients	0	10	2	1	50	YES
THP 11	5	In-patients & Out-patients	3	5	1	1	100	YES
THP 12	3	In-patients & Out-patients	12	50	5	4	100	YES
THP 13	18	In-patients & Out-patients	2	20	3	1	100	YES
THP 14	22	In-patients & Out-patients	15	30	3	3	100	YES
THP 15	4	In-patients & Out-patients	14	30	2	1	100	YES
THP 16	37	In-patients & Out-patients	3	50	2	1	50	YES
THP 17	35	In-patients & Out-patients	3	5	1	2	50	YES
THP 18	37	In-patients & Out-patients	4	30	6	1	100	YES
THP 19	34	In-patients & Out-patients	14	30	1	1	100	YES
THP 20	20	In-patients & Out-patients	3	30	4	1	100	YES

### Key informants

To access communities and identify individuals with psychosis who would not present to either formal or informal providers, we engaged key informants who, because of their involvement and knowledge of the communities across the catchment area, may have known of such individuals. These key informants included:

- The twenty THPs we had engaged were also considered key informants due to their involvement with and location within the community.
- Clinic managers of CHCs and PHCs as well as the 187 CCGs described above and their 12 supervisors.
- Specific contact persons at all 10 NGOs and research organisations active in the region, the university health clinic, the prison health centre and the eight police precincts within Msunduzi (as well as three outside but neighbouring Msunduzi).
- Several Ward Councillors and members of Ward Committees including the so-called “War Rooms” of several wards. These were selected based on the availability and willingness of these individuals to participate. War Rooms comprise a range of representatives within the community, who meet to discuss issues and challenges arising in these communities. We were informed that an individual posing ‘challenging behaviour’ or potential risk in the community, would likely be mentioned in War Room meetings.

## Discussion

This is the first study to map the potential points of contact that people with psychosis may engage with in a highly diverse, predominantly low income, and well-defined urban, peri-urban and rural municipality in South Africa, where little has hitherto been known about the extent to which formal, informal and popular modes of care delivery can be incorporated into a surveillance infrastructure for reliably estimating the incidence of psychotic disorders in novel settings in the Global South. Building on work piloted in our INCET study [17, 38], and adapting the INTREPID methodology [9], we were able to identify and engage with providers from both formal (health service) and informal (folk and other) sectors within Msunduzi during the mapping phase of our study. We were successful in establishing a productive collaboration with a network of Traditional Health Practitioners across the catchment area – a key partnership in view of the important role played by THPs within communities and in the help-seeking practices of people with psychosis [40]. This endeavour paved the way for a reliable case ascertainment methodology in this context, underscores the critical importance of detailed mapping of catchment areas prior to conducting epidemiological fieldwork of severe mental illnesses in the Global South, and could be further adapted for similar studies in other parts of South Africa and beyond.

In the effort to generate a greater evidence base on psychosis in the Global South, it is essential to understand the social, economic and cultural context and identify and engage with all stakeholders and potential providers of care. This approach has relevance too for case surveillance within a public health framework and aligns well with the recently adopted Mental Health Policy and Strategic Plan framework (2023–2030) by the South African Department of Health, which explicitly lays out in its terms of reference for District Mental Health Teams the intention to “Adopt a public health approach to the mental health of the district, conducting a situation analysis of mental health needs and service resources in the district population …” [48]. In Gauteng province, such teams have been established to strengthen the district mental health services with the task of conducting such situational analyses and improving intersectoral collaboration towards early identification, treatment and collaborative care of people with serious mental illness including psychosis [49]. Importantly then, in this approach, all participants in the case surveillance infrastructure are also considered partners in creating future collaborative care services for people with psychosis and other severe mental disorders.

Until recently, there was very little data available from representative samples of first-episode or untreated psychosis from low- and middle-income countries, especially on the African continent. The INTREPID study has begun to change that (and more recently the SCOPE study in Ethiopia [50]) and, by publishing detailed accounts of its methodology, and in particular, its system for mapping catchment areas and establishing structures for identifying cases, INTREPID provided a template for other studies to use in undertaking similar research in other diverse settings.

In PSYMAP-ZN, we set out to conduct a similar study to INTREPID, within a catchment area in South Africa, characterised by substantial geographical variation in levels of poverty/wealth, urbanicity/rurality, ethnic and cultural diversity, health and crime status. INTREPID had shown the critical importance of ‘mapping’ the catchment area for a deeper understanding of not just its population and geographical dynamics but also its formal and informal health resources. As discussed above, this exercise is not just important for research purposes, but also has relevance for a public mental health approach to surveillance of mental disorders within community settings. This paper reports on this mapping and surveillance exercise and its findings.

### Limitations

In undertaking what was a challenging and complex aim of identifying and engaging with providers of care and key informants across formal, informal and popular sectors of Msunduzi, we unsurprisingly encountered several limitations.

First, while we were confident that we identified all providers of care within the formal health sector, we cannot claim to have achieved this in the informal sector. For example, we were able to identify most Christian, Muslim and Hindu places of worship, but there were almost certainly smaller religious groupings that we missed. A high proportion of the South African population identifies as religious, and we know, for example, that within those identifying as Christian, there is a wide array of non-denominational groupings. In an area as big as Msunduzi, it would be difficult to identify every such grouping. Likewise, we know that we did not identify and engage with all THPs in the region. The reasons for this have been elaborated above and include various complex political and organisational issues beyond our control. However, as explained earlier, we do feel confident that we engaged most of those from across the region who were recognised and accredited by local traditional authorities and who were known to treat people with mental health presentations. Nevertheless, our inability to engage all THPs who might encounter people with untreated psychosis, must be acknowledged as an important limitation for case-ascertainment in PSYMAP-ZN. We know THPs operate in both urban and rural areas and we are likely to have missed some in both these settings. Thus, it is difficult to estimate how this would impact under-ascertainment in rural versus urban settings and thereby impact potential rural-urban differences in incidence. This underscores the importance of seeing the development of an infrastructure to provide a reliable epidemiology of psychotic disorders in this context as an ongoing, iterative process; investment in research programmes that establish such an infrastructure requires sustained, careful effort to lead to meaningful research that is given the potential to aid population mental health in these contexts in the long-term.

Second, we encountered barriers to engagement and participation in the referral system with some categories of providers, in both the formal and informal sectors. For example, there was one Pentecostal church which performed faith healing practices for people with mental illness, where our attempts at engagement were met with resistance, and we were unable to include them in the referral system. Also, while we were able to establish a referral and surveillance agreement with private practitioners, some difficulties were encountered, and this may have led to an underestimation of cases via these routes. However, based our clinical knowledge and experience within South Africa we also know that (a) very few people with FEP are treated privately, and (b) those that are treated, are generally referred quickly into the public system.

Finally, our 12 months of mapping for PSYMAP-ZN was interrupted by the Covid-19 pandemic, which meant the mapping study actually spanned a total period of about 18–20 months (with a period of very little activity possible in the middle). We were able to maintain to some extent relationships and engagement with providers we had identified and contacted prior to the onset of the Covid lockdown in South Africa. Some activities were very difficult during this period, for example, engagement with THPs necessitated face-to-face contact and so this was largely suspended for the 6–9 months of lockdown. When direct contact was again permitted, we were able to re-establish engagement with all of our 20 THP key informants.

## Conclusion

Despite these limitations, we are confident that we were able to map many key providers and informants in both formal and informal sectors within Msunduzi; and were able to establish a surveillance and referral system with the majority of those most likely to encounter people with new onset and untreated psychosis in the region. Specifically, we engaged all public primary and secondary mental health providers; all private psychiatrists and approximately 80% of private GPs and psychologists; all CCGs and their supervisors; most known religious institutions providing healing practices for people with mental health problems; and a group of THPs from across the region who were accredited and recognised by traditional structures as having expertise in treating people with mental health presentations.

In conclusion, we established a platform for the identification of a representative sample of people with untreated psychosis in the PSYMAP study in Msunduzi. This is important as the findings of PSYMAP will not only contribute new data on the epidemiology, clinical manifestation and aetiology of psychosis in diverse settings, but will also yield information on help-seeking behaviours, the needs of people with psychosis and their caregivers, and the types and extent of services required to meet these needs in South Africa [51]. In terms of the latter, it is clear that appropriate and effective service planning will require creative and innovative efforts to develop and implement a collaborative service model that incorporates both formal and informal providers of care [52]. If these objectives can even partially be met, this will go some way towards reducing the mental health treatment gap for people living with serious mental illnesses such as psychosis in the Global South.

## Data Availability

No datasets were generated or analysed during the current study.
